# Molecular profiling of urinary extracellular vesicles in chronic kidney disease and renal fibrosis

**DOI:** 10.3389/fphar.2022.1041327

**Published:** 2023-01-12

**Authors:** Melanie Tepus, Elisa Tonoli, Elisabetta A. M. Verderio

**Affiliations:** ^1^ Centre for Health, Ageing and the Understanding of Disease (CHAUD), School of Science and Technology, Nottingham Trent University, Nottingham, United Kingdom; ^2^ Department of Biological, Geological, and Environmental Sciences, Alma Mater Studiorum, University of Bologna, Bologna, Italy

**Keywords:** kidney, renal fibrosis, urinary extracellular vesicles (uEVs), biomarkers, chronic kidney disease (CKD), urine

## Abstract

Chronic kidney disease (CKD) is a long-term kidney damage caused by gradual loss of essential kidney functions. A global health issue, CKD affects up to 16% of the population worldwide. Symptoms are often not apparent in the early stages, and if left untreated, CKD can progress to end-stage kidney disease (ESKD), also known as kidney failure, when the only possible treatments are dialysis and kidney transplantation. The end point of nearly all forms of CKD is kidney fibrosis, a process of unsuccessful wound-healing of kidney tissue. Detection of kidney fibrosis, therefore, often means detection of CKD. Renal biopsy remains the best test for renal scarring, despite being intrinsically limited by its invasiveness and sampling bias. Urine is a desirable source of fibrosis biomarkers as it can be easily obtained in a non-invasive way and in large volumes. Besides, urine contains biomolecules filtered through the glomeruli, mirroring the pathological state. There is, however, a problem of highly abundant urinary proteins that can mask rare disease biomarkers. Urinary extracellular vesicles (uEVs), which originate from renal cells and carry proteins, nucleic acids, and lipids, are an attractive source of potential rare CKD biomarkers. Their cargo consists of low-abundant proteins but highly concentrated in a nanosize-volume, as well as molecules too large to be filtered from plasma. Combining molecular profiling data (protein and miRNAs) of uEVs, isolated from patients affected by various forms of CKD, this review considers the possible diagnostic and prognostic value of uEVs biomarkers and their potential application in the translation of new experimental antifibrotic therapeutics.

## 1 Introduction

### 1.1 Extracellular vesicles

“For some years the small dancing bodies in the plasma of the blood have proved a veritable mare’s nest to many workers. What are they? And where do they come from?” (Horder, 1899). Particles of the size and characteristics of extracellular vesicles (EVs) were first suggested as “blood dust” in 1899 (Horder, 1899). They were detected in blood initially (Johnstone et al., 1987) as “particulate material (platelet-dust)” that could be isolated by ultracentrifugation, high in phospholipid content and showing coagulant function (Wolf, 1967). In particular, the presence of “50-nm buds into the extracellular milieu” was reported during the *in vitro* maturation of sheep reticulocytes, recovered by centrifugation at 100,000xg. In these particles it was noted that the transferrin receptor faced the extracellular medium, according to the later consolidated notion that one membrane inversion occurs in the initial endocytosis and a second inversion in the stage of intra-vesicular budding, in the biogenesis of these vesicles. Although EVs were initially proposed to contain discarded products into the medium or fluids, it is now well established that EVs are small membrane-bound carriers of proteins, nucleic acids, and lipids. There are several types of EVs that differ in their origin, size, and content, classified as large apoptotic bodies, microvesicles (also called ectosomes), exosomes (Van Niel et al., 2018), and smallest exomeres (Zhang H et al., 2018). Exosomes, which are 40–100 nm in size, are intraluminal vesicles that are formed within the endosomes, the multivesicular body (MVB), by the inward budding of endosomal membrane and are released after the fusion of endosome with the plasma membrane. The mechanisms of exosome biogenesis are complex processes that vary according to the cell type, stimuli received by the cells and the cargo. Sorting of the cargo into exosomes and exosome secretion involves subunits of endosomal sorting complex required for transport (ESCRT) machinery, consisting of four protein complexes (ESCRT-0, -1, -2, -3), as well as ESCRT-associated proteins such as apoptosis-linked gene-2-interacting protein X (ALIX) and tumor susceptibility gene 101 (TSG101). However, cargo clustering and membrane budding can occur in both ESCRT-dependent and ESCRT-independent manners (Bruno et al., 2016; Hessvik and Llorente, 2017). Ectosomes, on the other hand, are 100–1000 nm in size and their biogenesis is based on outward budding at the plasma membrane from where are released in the extracellular space (Heijnen et al., 1999). As both categories of EVs (exosomes and ectosomes) are recovered in the extracellular environment and there is no consensus on endosomal or ectosomal markers to track their origin, the International Society for extracellular vesicles has proposed to refer to them according to their size, as simply “small extracellular vesicles” and “large extracellular vesicles” (Théry et al., 2018).

### 1.2 Chronic kidney disease and renal fibrosis

In chronic kidney disease (CKD) the essential kidney functions progressively decrease, with irreversible alterations of the kidney structure. The leading causes of CKD are recognized as being diabetes and hypertension (Couser et al., 2011) but many kinds of kidney injuries and loss of functional nephrons can induce and accelerate the pathology.

CKD is a growing public health issue with an estimated prevalence of 8%–16% worldwide (Bikbov et al., 2020). It is reported to be the 12th largest global cause of death, either as a primary condition or due to secondary causes such as stroke or cardiovascular disease. The number of people with CKD reached 700 million in 2017. Many of these patients are often asymptomatic until they progress to an advanced stage. Adequate screening and early treatment of renal disorders prevent the onset of CKD and applied disease management strategies reduce the progression to end-stage kidney disease (ESKD). However, when CKD progresses to ESKD, dialysis and kidney transplantation are the only options left. Renal replacement techniques are lifesaving, but also very costly treatments, as a consequence, many low-income countries are in shortages of such services, resulting in premature deaths (Bikbov et al., 2020; Jha et al., 2013).

In 2002, the National Kidney Foundation guidelines classified CKD into five stages according to estimated glomerular filtration rate (eGFR) levels (mL/min/1.73 m^2^). A decade later, the guidelines further detailed stage 3 into stages 3a and 3b (Table 1) recommending a classification of CKD based on both eGFR and albuminuria (Pavkov et al., 2018). Moreover, albuminuria levels, either albumin excretion rate (AER) or albumin-to-creatinine ratio (ACR), are distinguished into three stages (Table 2) (Inker et al., 2014).

**TABLE 1:** Classification of CKD based on estimated glomerular filtration rate (eGFR).

eGFR (ml/min/1.73 m^2^)	
Stage 1	≥90
Stage 2	60–89
Stage 3a	45–59
Stage 3b	30–44
Stage 4	15–29
Stage 5	<15

**TABLE 2:** Classification of CKD based on albuminuria measured as albumin excretion rate (AER) or albumin-to-creatinine ratio (ACR).

	Albuminuria		
	AER (mg/24 h)	ACR (mg/g**)**	ACR (mg/mmol)
Stage A1	<30	<30	<3
Stage A2	30–300	30–300	3–30
Stage A3	>300	>300	>30

ACR is calculated by dividing albumin concentration in milligrams with creatinine concentration in grams. To express ACR in mg/mmol, mg/g values are multiplied by the conversion factor of .113, based on the molar mass of creatinine.

A number of nephrotic syndromes and renal conditions can lead to the development of CKD and are associated with increased rate of mortality and morbidity (Kolb et al., 2021), from glomerulonephritis, to inherited conditions and auto-immune diseases. Diabetic nephropathy (DN) is the most common cause of ESKD, and autosomal dominant polycystic kidney disease (ADPKD) is one of the most common genetic kidney diseases. The topic of CKD types is discussed along urinary EVs (uEVs) biomarkers in Section 2. In addition to determined aetiologies, widespread environmental toxins, such as lead (Skerfving and Bergdahl, 2007), pose a risk for the development of CKD. Increased blood levels of lead are associated with decreased eGFR and increased odds of developing CKD, especially in patients aged ≥60 (Spector et al., 2011). Dietary factors such as phosphorus and protein can be detrimental to the development and progression of CKD. Phosphaturia induces tubular injury, inflammation, interstitial fibrosis and reduced renal Klotho, a coreceptor for the hormone fibroblast growth factor 23 (FGF23) necessary to reduce the renal phosphate reabsorption and thus maintain normal serum phosphate levels (Gutierrez et al., 2005; Urakawa et al., 2006; Santamaría et al., 2018). Several drugs have been associated with renal damage and increased CKD incidence, including for example, antiretroviral drugs used for HIV treatment such as tenofovir disoproxil fumarate, indinavir, and atazanavir (Kopp et al., 1997; Mocroft et al., 2010). Excessive intake of anabolic steroids can lead to hepatotoxicity and glomerulosclerosis, linked to the development of glomeruli hypertrophy and podocytes loss in response to body mass increase (Herlitz et al., 2010). Other examples are that of chemotherapy drugs (e.g., cisplatin and methotrexate) or non-steroidal anti-inflammatory drugs, which are known to induce acute kidney injury (reviewed by Izzedine and Perazella, 2017). Furthermore, nephrotoxicity might also be promoted by alterations in the mechanisms of renal excretion of certain drugs *via* specific transporters located on the basolateral and apical membranes of renal TECs, such as organic cation/anion transporters (OCT and OAT respectively) and efflux transporters (e.g., multidrug resistance proteins and P-glycoprotein) (Izzedine and Perazella, 2017).

Renal fibrosis has been recently linked to disorders of lipid metabolism, with abnormal accumulation of lipids in the kidney tissue and dysregulation of signaling pathways (reviewed in Yuan et al., 2022). Extensive accumulation of lipid droplets in TECs, podocytes, mesangial cells and fenestrated endothelial cells is observed in DN patients (Herman-Edelstein et al., 2014), accompanied by downregulation of acyl-CoA oxidase 1 (ACOX1), carnitine palmitoyltransferase 1 (CPT1), and peroxisome proliferator-activated receptors (PPAR-α and -β), and upregulation of LDL receptor and fatty acid transporter CD36 (Herman-Edelstein et al., 2014). Genome wide unbiased transcriptome analysis confirmed downregulation of critical fatty acid oxidation (FAO) enzymes and regulators in the fibrotic kidney (Kang et al., 2015).

**FIGURE 1:**
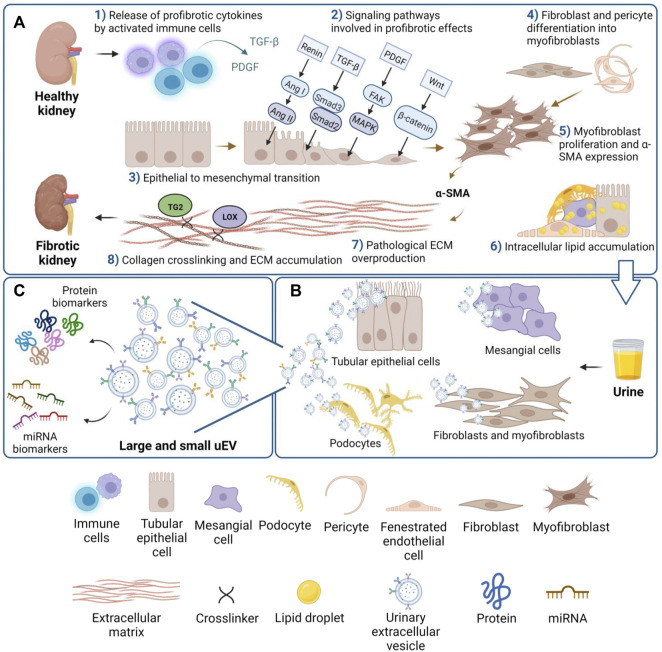
Overview of the main cell and tissue changes leading to kidney fibrosis. **(A)** Schematic view of eight of the main events involved in fibrosis: activation of cells and release of cytokines (1); activation of profibrotic signaling pathways (2); EMT (3); differentiation of mesenchymal cell types to activated myofibroblasts (4); α-SMA expression (5); accumulation of lipids drops in renal cells (6); ECM synthesis and overproduction (7); collagen crosslinking (8). **(B)** uEVs secreted from specific renal cell types can be collected in urine, and **(C)** the uEVs cargo of protein and miRNA likely mirrors the fibrotic state.

Renal fibrosis is the common underlying cause of all progressive kidney diseases, and the final manifestation of CKD as it progresses to ESKD. Renal fibrosis may be defined as unsuccessful wound-healing of kidney tissues which have undergone chronic and sustained injury. A dynamic system, renal fibrosis involves extracellular matrix (ECM) components and renal and infiltrated cell types. This topic has been extensively reviewed in the past decade, for example by Webster et al., 2017; Boor et al., 2010. As schematically shown in Figure 1, renal fibrosis is characterized by excessive deposition of connective tissue in the kidney parenchyma, in concert with the process of epithelial to mesenchymal transition (EMT) of tubular epithelial cells (TEC) into myofibroblasts, the key mediators in the remodeling of fibrotic tissue. EMT is a highly orchestrated process involving loss of epithelial cell adhesion, *de novo* α-smooth muscle actin (α-SMA) expression, disruption of tubular basement membrane and increased cell migration and invasion (Yang and Liu, 2001).

Activation of myofibroblasts is a common manifestation of fibrosis in multiple organs, but their origin is different. In renal fibrosis, fibroblasts, together with pericytes acquire the phenotype of myofibroblasts. These scar-forming cells increasingly produce ECM components and express α-SMA (Kuppe et al., 2021). It has been reported that half of the accumulated myofibroblasts arise from the local proliferation of resident fibroblasts, while the other half originates from either the differentiation of bone marrow-derived mesenchymal stem cells or through cell transformations like EMT or endothelial-to-mesenchymal transition (EndMT) (LeBleu et al., 2013). In liver fibrosis, the predominant source of myofibroblasts are hepatic lipocytes that synthesize and secrete collagen and are therefore considered precursors of fibroblast-like cells in pathological fibrosis (Friedman et al., 1985). In cardiac fibrosis, in addition to α-SMA-expressing myofibroblasts, cartilage intermediate layer protein 1 (CILP-1)-fibroblasts and thrombospondin-4 (THBS4)-fibroblasts have emerged as predominant fibrogenic populations (McLellan et al., 2020). Pulmonary mesenchymal cells, on the other hand, are very heterogeneous with two main mesenchymal lineages identified, namely mesenchymal alveolar niche cells that support alveolar growth and regeneration and axin2+ myofibrogenic progenitor cells, which contribute to pathologically damaging myofibroblasts after injury (Zepp et al., 2017).

The cytokine transforming growth factor-beta (TGF-β) is the major modulator of EMT and a main inducer of profibrotic events in multiple organs *via* the SMAD pathway (Clouthier et al., 1997; Sime et al., 1997; Deten et al., 2001). By mediating the migration of mesenchymal fibroblasts to the adjacent interstitial parenchyma TGF-β enhances ECM production and accumulation, thus contributing to the deterioration of renal function (Sato et al., 2003). TGF-β signaling interacts with other cell signaling pathways such as the renin-angiotensin-aldosterone system (RAAS), a critical pathway leading to CKD, in which renin promotes production of angiotensin I (Ang I), which is converted to Ang II by angiotensin-converting enzyme (ACE) (AlQudah et al., 2020). Treatment of mesangial cells with Ang II, a pro-fibrotic effector of RAAS, induces the synthesis and secretion of TGF-β, increasing ECM components such as biglycan, fibronectin, and collagen I, in glomerulosclerosis (Kagami et al., 1994). Ang II was found to induce renal inflammatory injury and fibrosis through binding to its well-known receptor AT1R (Zhang et al., 2015) and to myeloid differentiation protein-2 (MD2) (Xu et al., 2017). Despite the classical RAAS pathway of fibrosis, there is an alternative RAAS pathway in which ACE2 is key to catalyze Ang II to Ang I-VII conversion, counteracting the pathological effects of the classical pathway (Liu et al., 2012). Furthermore, Ang IV attenuates diabetic fibrosis and myocyte apoptosis as observed in cardiac tissue (Zhang et al., 2021). Another key mediator of fibrosis development, platelet derived growth factor (PDGF), induces mitogen-activated protein kinase (MAPK) signaling *via* focal adhesion kinase (FAK) activation promoting vast fibroblasts and myofibroblasts proliferation and ECM synthesis (Li et al., 2000; Andrae et al., 2008). All of these pro-fibrotic cytokines and growth factors (such as TGF-β, PDGF) are produced by activated leukocytes and macrophages that accumulate in the glomerulus and interstitium of fibrotic tissue (Duffield, 2014).

A key enzyme that has a pathological role in renal scarring is transglutaminase 2 (TG2), a member of a large family of enzymes responsible for Ca^2+^ dependent transamidation, which is regarded as the most abundant member in kidney (Burhan et al., 2016). TG2 is a recognized marker of kidney fibrosis progression, by cross-linking and stabilizing the ECM, but also by activating TGF-β *via* a heparan sulfate/syndecan-4 interaction (Figure 1) (Scarpellini et al., 2014; Verderio et al., 2016; Burhan et al., 2016). TG2 is externalized in experimental models of CKD and found secreted in urine (Da Silva Lodge et al., 2022) and in urinary vesicles (Furini et al., 2018). Another group of ECM cross-linking enzymes whose increased activity is associated with tissue fibrosis is the lysyl oxidase (LOX) family, which has been reported in hepatic (Siegel et al., 1978), pulmonary (Counts et al., 1981), and renal fibrosis (Goto et al., 2005). A recent study on human samples found increased serum LOX levels in patients with renal fibrosis, thus suggesting serum LOX as a diagnostic marker for renal fibrosis (Zhang et al., 2020; Zhang X et al., 2022).

Renal fibrosis correlates with loss of renal function, which is especially evident in the pathology of diabetic nephropathy (DN) (Qian et al., 2008). The outcome of CKD may vary among individuals and is linked to fibrosis progression. Three forms of CKD can be distinguished based on how it develops over the years: “stable CKD” when patients maintain stable eGFR levels, “reversal CKD” when eGFR levels improve and “progressive CKD” when eGFR levels irreversibly decrease over the years, a stage reached by majority of CKD patients (Zhong et al., 2017). In addition to eGFR and albuminuria, which are the most common tests used in clinical practice, additional screening tests such as measurements of proteinuria, serum creatinine and/or cystatin C and serum urea may be used. There is not a perfect test as each of these approaches have some limitations, since age, body size, gender, muscle mass, as well as different disease aetiologies can affect their sensitivity and specificity (Rysz et al., 2017). Kidney biopsy is the gold standard for diagnosing and determining the severity of CKD. It is the best test of fibrosis although intrinsically imperfect being an invasive and biased procedure, not always specific to a particular pathology (Corapi et al., 2012; Bleyer et al., 2017). Detection of renal fibrosis often means diagnosis of CKD and several markers of CKD and fibrosis have been proposed (Lopes et al., 2019). However, as these markers are hard to trace in biofluids, there is a raising interest in biomarkers which are cargo of urinary EVs, where biomarkers are concentrated and protected thus potentially more accessible to test the onset of fibrosis and CKD progression.

### 1.3 Urine as a source of biomarkers

Urine is an ideal source of biomarkers, since it contains cellular elements, biochemicals and proteins that are filtered through the glomeruli, but are also derived from renal tubule excretion and urogenital tract secretion, all of which can reflect potentially a physio-pathological state. Virtually all types of kidney cells are identifiable in urine (podocyte, proximal tubule, loop of Henle, and collecting duct), but also macrophages, lymphocytes, and bladder cells, as recently validated by single-cell transcriptomic of urine samples (Abedini et al., 2021).

Moreover, urine is easily accessible in large volumes, and it is collected in a non-invasive way (Harpole et al., 2016). A number of urinary proteins have been proposed as biomarkers of kidney damage in kidney disease progression, such as uromodulin (Prajczer et al., 2010), kidney injury marker 1 (KIM1) (van Timmeren et al., 2007), and neutrophil gelatinase-associated lipocalin (NGAL) (Viau et al., 2010). Furthermore, microRNAs (miRNAs), small ∼22 nucleotide long RNA molecules that play a key role in posttranscriptional gene regulation, have also been reported to correlate with CKD, for example miR-126, miR-155, miR-29b (Beltrami et al., 2018), miR-1228-3p, and miR-27b-3p (Conserva et al., 2019), and miR-196a (Zhang C et al., 2018). The miRNA biogenesis is a multi-step process that starts in the nucleus from non-protein monocistronic or polycistronic coding genes (Bartel, 2004). Following transport of pre-miRNA to the cytoplasm, mature and functional miRNAs are generated and incorporated in the RNA-Induced Silencing Complex (RISC) to target messenger RNA (mRNA) to the 3’ untranslated region (Bernstein et al., 2001). Target mRNAs are degraded by endonuclease Argonaute (RISC complex), or their expression repressed (Wilson and Doudna, 2013). Deregulation of miRNAs is linked with renal fibrosis and CKD (Li et al., 2013; Macconi et al., 2012; Lv et al., 2018).

Because urine allows testing of kidney health, it is of great interest to establish whether urinary molecules can be detected in a sensitive and specific way to assist in early diagnosis and prognosis of kidney disease. As the majority of urinary proteins such as albumin and IgG are filtered from the blood, they are qualitatively similar to the abundant plasma proteins (Spahr et al., 2001). Being highly concentrated in pathology, they greatly interfere with the detection of other urinary rare biomarkers (Filip et al., 2015), limiting dramatically the sensitivity of the approach. Instead, EVs which can be isolated from urine and mirror cell changes in pathology may offer an alternative sensitive platform for biomarkers discovery. Therefore, EVs molecules may better meet the definition of “good biomarker” (Bennet and Devarajan, 2017).

### 1.4 Urinary extracellular vesicles

In urine, “membrane-bound vesicles” with procoagulant activity were firstly reported in a 100,000xg precipitate and characterized as 100 nm to 1.1 micron particles by scanning electron microscopy (Wiggins et al., 1986). After initial observations in normal urine, analysis of urine specimens of patients with renal injury led to the discovery that the proximal tubule sheds enzymes from the brush border into urine early post-injury, and in particular patients with glomerulonephritis were found to excrete “blebs” of 100–300 nm. Glomerular podocytes were reported to release vesicles coated by complement receptor-1, which was proposed as a possible marker for podocyte injury (Pascual et al., 1994). An advanced first characterization of EVs in human urine (uEVs) was first reported in 2004 (Pisitkun et al., 2004). Here uEVs were isolated by an ultracentrifugation protocol and investigated by immunogold electron microscopy. Proteomic profiling of uEVs led to the characterization of the protein cargo, showing the presence of endosomal pathway proteins and providing an explanation of how certain proteins such as aquaporin-2 (AQP2) are released in urine. Therefore, the idea that uEVs could be a tool for disease biomarker discovery started to form. We know now that uEVs can be released by cells from kidney, bladder, and the urogenital tract in general (reviewed by Erdbrügger and Le, 2016), and although urine may contain some of circulating EVs from serum, the majority of uEVs are thought to originate from renal cells (as schematically illustrated in Figure 1B). The reason may be in the mechanical and charge barrier of the glomerulus, which would prevent serum EVs from easily passing through under physiological conditions (Pisitkun et al., 2004; Gildea et al., 2014).

Small and large uEVs are believed to have analogous functions to circulating EVs in mediating cell-to-cell communication and intercellular signal transmission, and their cargo is expected to reflect physiological and pathophysiological conditions of the renal cells (Gildea et al., 2014; Street et al., 2011). Recently new nanoparticles, denominated exomeres have been identified through asymmetric-flow field-flow fractionation, a separation technique based on hydrodynamic size, which has brought to their discovery (Zhang H et al., 2018). Their size is about 35 nm making exomeres the smallest type of extracellular particles compared to large and small EVs. Although there is evidence that exomeres carry functional cargo too and thus play a role in cellular communication (Zhang et al., 2019), there are no full reports yet of exomeres in the renal system.

Apart from carrying and delivering their cargo to target cells, EVs have the ability to change the phenotype of the target cells by transferring different populations of proteins and nucleic acids (Quesenberry et al., 2014). This makes them a powerful mechanism of disease spreading. For example, it has been demonstrated that TGF-β-containing EVs secreted from injured epithelial cells, after their uptake by target cells, can stimulate profibrotic effects such as promoting cell proliferation, type I collagen production and α-SMA and F-actin expression on the same cell type from which they are secreted (Borges et al., 2013). On the other hand, EVs released from different cell types such as mesenchymal stem cells have shown renal protective and pro-survival effects in acute kidney injury both *in vitro* and *in vivo* once uptaken by TECs (Bruno et al., 2012). Another example is provided by CD133, a glycoprotein also known as prominin-1 and marker of kidney progenitor cells involved in tissue repair. CD133-containing EVs released by this population of stem cells have been shown to favor renal regeneration after injury (Ranghino et al., 2015). Given these mounting discoveries, there is a great interest in the detection of proteins and nucleic acids isolated from uEVs for potential application as diagnostic, prognostic, and therapeutic biomarkers for CKD (Cocucci and Meldolesi, 2015; Zhang et al., 2016a), a condition which still relies on invasive tools to establish renal fibrosis.

### 1.5 Tracking the cell origin of urinary extracellular vesicles

**FIGURE 2:**
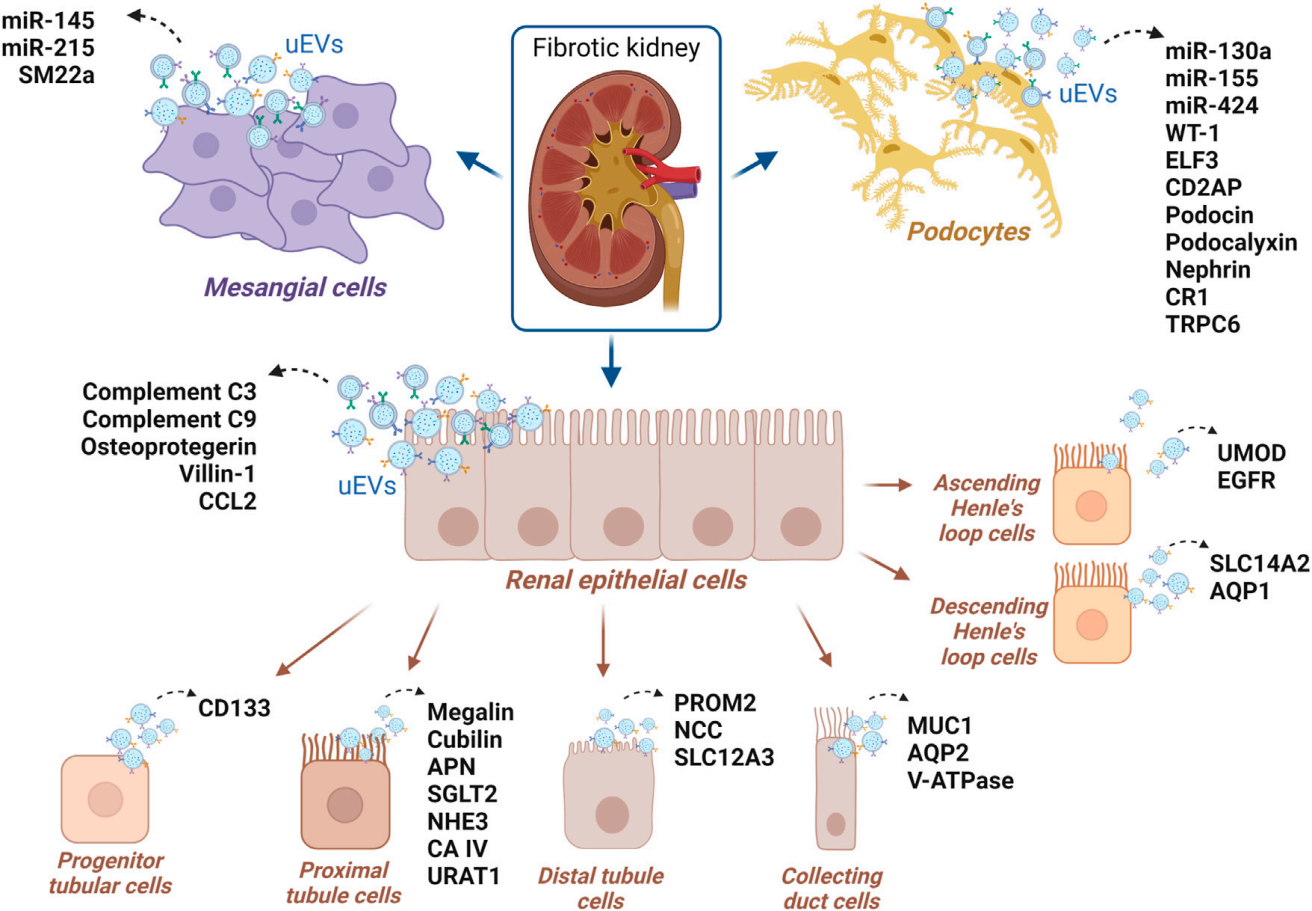
uEVs proteins and uEVs miRNAs suggested as potential markers of CKD, for which the renal cell origin has been reported in the literature.

Extracellular vesicles are not all the same and they can be classified based on differences in size, however, what ultimately gives them an identity is their cargo. The most well-established vesicular markers are tetraspanins (CD9, CD63, CD37, CD81, and CD82), the most abundant proteins in the membrane of small EVs, with roles in cell adhesion, motility, membrane fusion, protein transport and signaling (Andreu and Yáñez-Mó, 2014). TSG101, ALIX, and clathrin are EVs markers associated with MVB biogenesis while annexins and small GTPases retinoic acid-binding (Rab) proteins are implicated in membrane transport, docking and fusion with target cells. Other markers involved with exosomal biogenesis are heat shock proteins Hsp70 and Hsp90 (Reddy et al., 2018). Membrane receptor integrins and lipid raft flotillins are also prominent markers (Merchant et al., 2017; Cricrì et al., 2021). Apart from generic markers, EVs display a cargo specific to the cell type from which they originate (Figure 2), and it is this which should enable tracking the cell origin of isolated EVs and therefore the cell type undergoing the pathological change. The International Society of Extracellular Vesicles (ISEV) has recently summarized protein markers specific to diverse cell types and segments within the kidney, upon evaluations by flow cytometry and Western blotting (reviewed by Erdbrugger et al., 2021). TECs are a source of CD24-containing uEVs, also known as cluster of differentiation-24 or heat stable antigen CD24. The presence of uEVs from podocytes may be determined by podocin, podocalyxin (PCLP1), nephrin, Wilms’ tumor-1 (WT 1), complement receptor-1 (CR1), and canonical transient receptor potential-6 (TRPC6). Urinary EVs from proximal tubular cells typically carry megalin, cubilin, aminopeptidase-N (APN), sodium/glucose cotransporter-2 (SGLT2), carbonic anhydrase (CAIV), Na^+^/H^+^ exchanger isoform-3 (NHE3) as well as urate transporter-1 (URAT1) (Turco et al., 2016). To identify uEVs from descending limb of Henle’s, solute carrier family-14 member 2 (SLC14A2) and aquaporin-1 (AQP1) may be utilized, while uromodulin and epidermal growth factor receptor (EGFR) should mirror the ascending limb origin, type-2 Na^+^-K^+^-2Cl^-^ cotransporter (NKCC2) the Henle’s loop (Turco et al., 2016). Moreover, it was commented before that progenitor tubular cells secrete EVs displaying CD133 (Prominin-1) (Erdbrugger et al., 2021). Collecting duct cells uEVs are marked by the presence of mucin-1, a glycoprotein that activates protective pathways in TECs after hypoxia in acute kidney injury (Pastor-Soler et al., 2015), but also AQP2 and V-ATPase (Turco et al., 2016). Distal tubule-derived uEVs typically display prominin-2, thiazide-sensitive Na-Cl cotransporter (NCC), and solute carrier family 12 member 3 (SLC12A3) (Pomatto et al., 2017; Erdbrugger et al., 2021; Turco et al., 2016). Beta-1 adrenergic receptor (β-1 AR) was reported as an uEVs marker of juxtaglomerular cells, and transgelin (SM22 alpha) of mesangial cell origin, while claudin-1 and cytokeratin eight are uEVs markers of parietal cell origin (Bowman’s capsule) and cytokeratin-19–20 of the transitional epithelium (renal pelvis) (Turco et al., 2016). Some uEVs markers are shared by more than one cell-type, especially ACE which was detected in uEVs from glomerulus and proximal tubules, AQP1 in uEVs from proximal tubules and Henle’s loop, and AQP2 found in the uEVs from distal tubules and collecting duct (Erdbrugger et al., 2021). Although there are still no known specific EVs markers for fibroblasts, PDGFRβ and CD73 which are highly expressed in the plasma membrane of fibroblasts, are employed as markers for respectively interstitial and cortical fibroblasts, despite also recognizing pericytes or proximal tubular cells (Asada et al., 2011; Schiessl et al., 2018; Perry et al., 2019; Arai et al., 2021). Recently, naked cuticle homolog 2 (NKD2) has been suggested as a marker of myofibroblasts and a potential therapeutic target, as its knockdown markedly reduces the expression of ECM components independently of TGF-β (Kuppe et al., 2021). NKD2 is a negative regulator of Wnt/β-catenin signaling, another pathway involved in fibrosis development, which is stimulated by TGF-β and plays a role in fibroblast activation and ECM synthesis (Akhmetshina et al., 2012).

## 2 Candidate markers of CKD types in urinary extracellular vesicles

### 2.1 Diabetic nephropathy

Diabetic nephropathy (DN), also termed diabetic kidney disease (DKD), is a type of glomerulonephritis usually characterized by the presence of persisting elevated albuminuria, diabetic renal lesions, and decreased eGFR in diabetic patients (Alsaad and Herzenberg, 2007). Multiple sections of the kidney undergo structural alterations such as thickening of the glomerular basement membrane (GBM), capillary and tubular basement membrane, as well as loss of endothelial fenestrations, mesangial matrix expansion, and drop in podocyte (Alicic et al., 2017). Podocytes can be functionally and structurally injured early in DN pathology, and a decrease in their number and density is associated with the development of proteinuria and DN progression (Su et al., 2010). Albuminuria is a strong predictor, however DN can also present low levels of albuminuria or albuminuria can regress (Kramer et al., 2003). Being driven by a rather heterogeneous array of pathways, from inflammation to tubular injury, the identification of patients progressing to ESKD is difficult and much effort has been placed to discover urine and plasma biomarkers of high-risk disease.

Apart from persistent albuminuria, DN is characterized by renal lesions, which are shown to be very heterogeneous in renal biopsies from type 2 diabetes (T2D) patients (Gambara et al., 1993). Moreover, DN in both insulin-dependent (Parving et al., 1988) and non-insulin-dependent (Parving et al., 1992) diabetic patients can be manifested by proliferative retinopathy and blindness, neuropathy, arterial hypertension, and is associated with increased morbidity and mortality. Monitoring DN through plasma or urine protein fingerprint has been exploited *via* a variety of proteomics approaches although hampered by the abnormal albumin level found in this condition (Van et al., 2017). Tubular injury marker kidney injury molecule-1 (KIM-1), traceable in blood and urine, has been proposed to lead to DN progression (Sabbisetti et al., 2014; Schrauben et al., 2021; Vaidya et al., 2011). Another tubular injury marker is N-acetyl-β-D-glucosaminidase (NAG) which has been linked with DN in patients with type-1 diabetes (T1D) (Vaidya et al., 2011), while haptoglobin emerged as a strong predictor of DN in patients with T2D (Bhensdadia et al., 2013). Meta-analysis of urinary liver-type fatty acid-binding protein (L-FABP) has shown potential of L-FABP to detect all stages of DN and predict disease severity and progression in patients with either T1D or T2D (Zhang L et al., 2022). A protein fingerprint of DN progression has been proposed by the Chronic Renal Insufficiency Cohort (CRIC) Study (Schrauben et al., 2021).

Growing attention has been paid to the relationships between uEVs cargo and DN detection and progression. The findings are summarized in Table 3 and include early and recent studies.

**TABLE 3:** Proteins isolated from uEVs, proposed to be biomarkers of CKD.

CKD etiology	uEV protein	Expression	Sample size	Method of analysis	Source
DN	Dipeptidyl peptidase-4 (DPP IV)	DN ↑	Normoalbuminuria, *n =* 43; Microalbuminuria, *n =* 50; Macroalbuminuria, *n =* 34	ELISA	Sun et al*.* (2012), *Diab Vasc Dis Res*
DN	Wilms’s tumor 1 transcription factor (WT1)	DN ↑	T1D, *n =* 48; Control, *n =* 25	Immunoblotting	Kalani et al. (2013), *PLoS ONE*
DN	α-microglobulin/bikunin precursor (AMBP)	DN ↑	DN, *n =* 5; Control, *n =* 5	LC-MS/MS + SRM	Zubiri et al. (2014), *Journal of Proteomics*
	Histone-lysine N-methyltransferase (MLL3)				
	Voltage-dependent anion-selective channel protein 1 (VDAC1)	DN ↓			
DN	Gelatinase	DN ↓	DN, *n =* 82	Fluorometric assay using FITC-gelatin as a substrate	Gudehithlu et al. (2015), *Am J Nephrol*
	*Ceruloplasmin			Sandwich ELISA	
DN	Regucalcin	DN ↓	DN, *n =* 4; Control, *n =* 3	Immunoblotting	Zubiri et al. (2015), *Transl Res*.
DN	Cathepsin A, C, D, L, X/Z/P	DN ↑	DN, *n =* 37; Control, *n =* 12	Proteome profiler human protease and inhibitor array kit	Musante et al. (2015), *J Diabetes Res*
	Kallikrein 13				
	Proteinase 3				
	Cystatin B				
DN	C-megalin	T2D ↑	T2D, *n =* 33; Control = 11	Immunoblotting	De et al. (2017), *Diabetes*
DN	Epithelium-specific transcription factor (ELF3)	DN ↑	DN, *n =* 25; MCD, *n =* 25; Control, *n =* 5	Immunoblotting	Sakurai et al. (2019), *PLoS ONE*
DN	CD73	DN ↑	DN, *n =* 10; Diabetics, *n =* 48; Control, *n =* 10	Immunoblotting	Cappelli et al. (2020), *Biochim Biophys Acta Mol Basis Dis*
DN ADPKD	Osteoprotegerin (OPG)	DN and ADPKD ↑	CKD, *n =* 14; Control, *n =* 4	LC-MS/MS + SRM QQQ-LC/MS	Benito-Martin et al. (2013), *PLoS ONE*
ADPKD	Periplakin	ADPKD ↑	ADPKD, *n =* 6; Control, *n =* 6	LC-MS/MS + Immunoblotting	Salih et al. (2016), *JASN*
	Envoplakin				
	Villin-1				
	Complement C3				
	Complement C9				
IgAN	*Ceruloplasmin	IgAN ↑	IgAN, *n =* 5; TBMN, *n =* 7; Control, *n =* 7	LC-MS/MS	Moon et al. (2011), *Proteomics*
	α-1-antitrypsin				
	Aminopeptidase N (APN)	IgAN ↓			
TBMN	Vasorin precursor	TBMN ↑			
IgAN	CCL2 mRNA	IgAN ↑	IgAN, *n* = 6; Control, *n =* 6	Real-time RT-PCR	Feng et al. (2018), *Am J Pathol*
IgAN MN FSGS LN	*Ceruloplasmin	MN, IgAN, FSGS, LN ↑	MN, *n =* 9; IgAN, *n =* 7; FSGS, *n =* 10; LN, *n =* 10; Control, *n =* 15	Sandwich ELISA	Gudehithlu et al. (2019), *Clin Exp Nephrol*
FSGS	Dipeptidase 1 (DPEP1)	FSGS ↓	CKD, *n =* 12; Control, *n =* 12	LC-MS/MS	Stokman et al. (2019), *Journal of Proteomics*
Nephronophthisis	Protocadherin Fat 4 (FAT4)	Nephronophthisis ↓			
CKD pool	Versican core protein (VCAN)	CKD ↑			
CKD pool	CD2AP mRNA	CKD ↓	CKD, *n =* 32; Control, *n =* 7	Real-time RT-PCR	Lv et al. (2014), *Clinica Chimica Acta*
CKD pool	CD133	CKD ↓	CKD, *n =* 12; Control, *n =* 7	Cytofluorimetric analysis	Dimuccio et al. (2020), *Am J Physiol Renal Physiol*

*recurrent in different studies; ↑ denotes increased and ↓ decreased expression compared to controls.

An initial investigation (Sun et al., 2012) was conducted on urine microvesicles obtained by capturing the microvesicles *via* a monoclonal antibody (AD-1) originally raised *versus* membrane-bound liver alkaline phosphatase from human liver cancer serum (Cheruvanky et al., 2007) and measuring dipeptidyl peptidase-IV (DPP IV or CD26) activity, an enzyme highly concentrated in the cortex. The rationale was that DPP IV is responsible for the degradation of active glucagon-like peptide-1 after meal intake, which in turn increases insulin secretion, thus it positively correlates with T2D. As expected, urinary microvesicle-DPP IV excretion was elevated in the T2D cohort compared with controls and positively correlated with ACR in diabetic patients with T2D, suggesting that urinary microvesicle-bound DPP IV could be linked with the severe DN.

Another early investigation on a larger cohort, this time based on uEVs isolated by a differential centrifugation method (Kalani et al., 2013) reported elevated levels of Wilm’s Tumor-1 (WT1) in the uEVs of T1D with proteinuria compared with patients without proteinuria, whereas in the non-diabetic control group WT1 expression was absent. WT1 is a zinc-finger transcription factor that plays a role in podocyte maturation and is used as a molecular marker of podocytes to assess podocyte lesions (Zhou et al., 2008; Su et al., 2010). It is believed that increased WT1 levels in uEVs can represent a decline in renal function in diabetics, since higher levels of exosomal WT1 were strongly associated with increased urinary protein excretion and with elevated serum creatinine and lower eGFR. Since urinary exosomal WT1 can be detected earlier than proteinuria and glomerular damage, WT1 may be regarded as a biomarker of early renal injury in diabetics (Kalani et al., 2013).

Work by Zubiri et al. (2014) identified three possible markers of DN in uEVs but in a small group of T2D patients with kidney disease. These were α-microglobulin/bikunin precursor (AMBP), a membrane glycoprotein that is expressed in liver and kidney; histone-lysine N-methyltransferase (MLL3), a protein involved in the methylation of Lys-4 of histone H3, a tag for epigenetic transcriptional activation (Cho et al., 2007); voltage-dependent anion-selective channel protein 1 (VDAC1), a channel on the outer membrane of mitochondria and plasma membrane (Lawen et al., 2005), which was found downregulated in the DN urinary exosomes, however, its presence in most cell types makes it a non-selective marker of kidney disease in DN.

In the streptozotocin (STZ)-induced rat model of early DN, differential proteomic gel electrophoresis analysis of the renal tissue extract revealed downregulation of the regucalcin protein (or senescence marker protein-30 (SMP30)), a protein involved in cellular calcium homeostasis and control of oxidative stress in DN. In a pilot study investigating regucalcin in the uEVs, the same trend was confirmed in DN rats and in humans (Zubiri et al., 2015).

Comparative proteomics between whole urine samples and urinary exosomes in a large study on patients with DN revealed exosomal ceruloplasmin and gelatinase as early biomarkers of kidney disease (Gudehithlu et al., 2015). Gelatinases, also known as matrix metalloproteinases, are proteins involved in the breakdown of ECM components such as collagen, laminin, elastin, and fibronectin. Their dysregulation is associated with pathological processes of fibrotic diseases, inflammation, and tumor progression (Thrailkill et al., 2007). Ceruloplasmin is an acute-phase plasma protein with various physiological roles such as copper carrying, facilitating transferrin-mediated iron transfer and uptake, plasma ferroxidase activity and scavenging free radicals; therefore, it is protective against tissue damage under inflammatory conditions (Arnaud et al., 1988). The two proteins were previously reported to be altered in the diabetic state (Thrailkill et al., 2007; Del Prete et al., 1997; Narita et al., 2006) so it is not surprising they have been pinpointed as uEVs proteins in DN. The levels of exosomal gelatinase and ceruloplasmin correlated with their changes in renal tissue, but not when considering their levels in the full urine, showing a better role of uEVs in mirroring the cell pathological phenotype. Therefore, gelatinase and ceruloplasmin could potentially be utilized as biomarkers of DN according to Gudehithlu et al. (2015). However, uEVs ceruloplasmin was not uniquely linked to DN as it emerged as a potential early biomarker of membranous nephropathy (MN), IgA nephropathy (IgAN), lupus nephritis (LN), and focal segmental glomerulosclerosis (FSGS). In all of these conditions, exosomal ceruloplasmin was consistently higher in CKD compared to control subjects (Gudehithlu et al., 2019). This was also confirmed in a longitudinal study performed on a passive Heymann nephritis (PHN) rat animal model, which is a homologous condition to human MN, showing that the level of urine exosomal ceruloplasmin increased significantly 1 week before the onset of proteinuria (Gudehithlu et al., 2019).

More recently, a study by Sakurai et al. (2019) uncovered induction of the epithelium-specific transcription factor ELF3 in the exosomes of podocytes cultured in a diabetic condition. ELF3 induction was inhibited by neutralization of the bone morphogenetic protein 4 (BMP4), which in turn regulates TGF-β receptor II - SMAD3 signaling (i.e., entrance of SMAD3 protein in the nucleus and transcription of proliferation genes) (Sakurai et al., 2019). Hence, a link between EVs ELF3 with TGF-β signaling was established, moreover exosomal ELF3 was also found to be specific for DN as present in uEVs DN patients and not in the uEVs of other CKD patients. Because it also correlated with eGFR decline, it was proposed as an early marker for podocyte injuries in DN.

Numerous studies have also identified microRNAs (miRNAs) biomarker candidates of uEVs origin in DN (Table 4). Early research in T1D patients with microalbuminuria versus normoalbuminuria and non-diabetic controls suggested the possibility to stratify T1D patients with and without kidney disease through uEVs. In particular, miR-130a, expressed by human podocytes, and miR-145, a marker of glomerular mesangial cells, were found to be enriched in urinary exosomes of patients with T1D who developed symptoms of microalbuminuria. miR-145 upregulation was confirmed *in vivo* in murine experimental models (diabetic/non-diabetic model) and mesangial cells *in vitro* (Barutta et al., 2013). Furthermore, urinary exosomal miR-155 and miR-424 were downregulated in incipient DN (Barutta et al., 2013). We know that miR-155 could participate in the inflammatory response in endothelial cells by targeting human angiotensin II type I receptor (hAT1R) (Louafi et al., 2010) and that miR-424 can bind to vascular endothelial growth factor (VEGF), receptor 2 (VEGFR2) and fibroblast growth factor receptor 1 (FGFR1), with reduction of endothelial cell proliferation, migration and morphogenesis (Chamorro-Jorganes et al., 2011). As these miRNAs are expressed by podocytes, their significantly reduced expression in podocyte-derived exosomes may be a useful indicator of early-stage DN (Barutta et al., 2013).

**TABLE 4:** miRNAs isolated from uEVs proposed as biomarkers of CKD.

CKD etiology	uEVs miRNA	Expression	Sample size	Method of analysis	Source
DN	*miR-155	DN (T1D) ↓	Microalbuminuria, *n =* 12; Normoalbuminuria, *n =* 12; Control, *n* = 10	Human TaqMan miRNA array A	Barutta et al. (2013), *Plos One*
	miR-424				
	miR-130a	DN (T1D) ↑			
	miR-145				
Early-stage DN	miR-192	DN (T2D) ↑	Normoalbuminuria, *n =* 30; Microalbuminuria, *n =* 30; Macroalbuminuria, *n =* 30; Control, *n =* 10	RT-qPCR	Jia et al. (2016), *J Diabetes Res*
	*miR-194				
	miR-215				
DN	miR-15b	DN (T2D) ↑	T2D albuminuric, *n =* 90; Normo-albuminuric, *n =* 46; Control, *n* 44	SYBR green custom miScript miRNA PCR Array	Eissa et al. (2016), *J Diabetes Complicat*
	miR-34a				
	miR-636				
DN	miR-320c	DN ↑	DN, *n =* 8; T2D, *n =* 8; Control, *n =* 8	Agilent microarrays	Delić et al. (2016), *Plos One*
DN	miR-877-3p	DN ↑	DN, *n =* 5; T2D, *n* = 5	miRCURY LNA array	Xie et al. (2017), *J Diabetes Res*
FSGS MCD	miR-1225-5p	MCD ↑	FSGS, *n =* 16; MCD, *n =* 5; Control, *n =* 5	MiRNA microarrays and TaqMan qRT-PCR	Ramezani et al. (2015), *Eur J Clin Invest*
	miR-1915	FSGS ↓			
	miR-663				
	*miR-155	FSGS ↑			
SLE	miR-335*	SLE ↑	SLE = 28; Control = 12	RT-qPCR	Perez-Hernandez et al. (2015), *Plos One*
	miR-302d				
	miR-200c				
	miR-146a				
LN	miR-26a	LN ↑	LN, *n =* 13; Control, *n =* 8	RT-qPCR	Ichii et al. (2014), *Plos One*
LNIV	miR-3135b	LNIV ↑	Active LNIV, *n =* 5; Active LNIV-CC, *n =* 5; Inactive LNIV, *n =* 4; Control, *n =* 3	miRNA high-throughput sequencing	Li et al. (2018), *J Biol Res (Thessalon)*
LNIV-CC	miR-654-5p	LNIV-CC ↑			
Renal fibrosis in LN	miR-21, miR-150, miR-29c	LN ↑ (miR-21, miR-150), ↓ (miR-29c)	LN, *n =* 45; Control, *n =* 20	miRCURY LNA Universal RT microRNA PCR	Sole *et al.* 2019), *Cells*
Paediatric NS	*miR-194-5P	Paediatric NS ↑	NS, *n =* 5 (pool of 25); Control, *n =* 4 (pool of 20)	High-throughput Illumina sequencing	Chen et al. (2019), *EBioMedicine*
	miR-146b-5p				
	miR-378a-3p				
	miR-23b-3p				
	miR-30a-5p				
IgAN	*miR-150	IgAN ↑	IgAN, *n =* 22; Control, *n =* 6	NanoString nCounter miRNA Expression Assay	Szeto et al. (2019), *BMC Nephrology*
	miR-204	IgAN ↓			
	miR-555				
Renal fibrosis	*miR-29	Renal fibrosis ↓	CKD, *n =* 32; Control, *n =* 7	RT-qPCR	Lv et al. (2013), *Am J Physiol Renal Physiol*
	miR-200				
CKD pool	miR-181a	CKD ↓	CKD, *n =* 15; Control, *n =* 10	Total RNA NGS	Khurana et al. (2017), *RNA*
CKD pool	*miR-21	CKD ↑	CKD, *n =* 41; Control, *n =* 5	RT-qPCR	Lange et al. (2019), *J Cell Mol Med*
CKD pool	*miR-21-5p	CKD ↑	DN (T2D), *n =* 14; DN (T2D) normal renal function, *n =* 15	PCR panels and individual PCR	Zang et al. (2019), *Sientific Reports*
	miR-30b-5p	CKD ↓			
CKD pool	miR-451	CKD ↑	CKD, *n =* 48; Control, *n =* 23	RT-qPCR	Kumari et al. (2020), *Front Physiol*

*recurrent in different studies; **↑** denotes increased and **↓** decreased expression compared to controls.

In a similar uEVs DN study but with a duplicated sample size, miR-192, miR-194, and miR-215 were found to be significantly increased in the microalbuminuric group of T2D-linked DN patients when compared to the normoalbuminuric group and healthy controls, but were decreased in the macroalbuminuric group (Jia et al., 2016). Furthermore, miR-192 and miR-215 expression levels significantly correlated with those of TGF-β1 in uEVs. Therefore, also these miRNAs are potential biomarkers in the early stages of DN, with miR-192 as a stronger candidate, having a markedly higher expression levels than the other two (Jia et al., 2016). The involvement of miR-192 in the development of DN has been previously reported in both *in vitro* and *in vivo* studies showing that its inhibition can result in a significant increase of its target Zinc Finger E-Box Binding Homeobox 1/2 (ZEB1/2), which in turn reduced the expression of collagen, TGF-β and fibronectin, known mediators of renal fibrosis (Putta et al., 2012). On the other hand, miR-194 has been found to play a role in renal ischemia-reperfusion injury, which often causes acute kidney injury and renal fibrosis, leading to renal failure. An *in vitro* study, performed on the human kidney proximal TEC line HK-2, showed that miR-194 expression was reduced in ischemia-reperfusion injury resulting in inhibition of HK-2 cell survival (Shen et al., 2018). When miR-194 was overexpressed using miR-194 mimics, the secretion of oxidative stress markers and pro-inflammatory cytokines was suppressed and HK-2 cell survival was improved through miR-194 binding and inhibition of its downstream target Ras homologue enriched in brain (RHEB) (Shen et al., 2018). miR-215 has been shown to be involved in mesangial cell phenotypic transition into myofibroblasts mediated by TGF-β. In particular, inhibition of miR-215 significantly reduced the mesangial cell phenotypic transition, whereas its overexpression enhanced it, by targeting catenin-beta interacting protein 1 (CTNNBIP1). CTNNBIP1 has a function in suppressing the Wnt/β-catenin signaling pathway that promotes the activation and upregulation of α-SMA and fibronectin, therefore, when miR-215 binds CTNNBIP1, the activated Wnt/β-catenin pathway freely contributes to the pathogenesis of DN (Mu et al., 2013).

Urinary exosomal miR-15b, miR-34a, and miR-636 belong to a unique genetic cluster and share functions related to renal diseases and diabetes mellitus, such as regulation of cell proliferation, apoptosis and cytokine release. An *in silico* analysis suggests that all three miRNAs share common target mRNAs involved in glucose homeostasis, angiogenesis and DN (Eissa et al., 2016). In a large T2D albuminuric study group, all three miRNAs were upregulated compared with normoalbuminuric patients and healthy control group (Eissa et al., 2016). However, in about 30% of normoalbuminuric patients, these miRNAs were also found increased. Their dysregulation correlated with age, BMI, hypertension, serum creatinine, glycosylated hemoglobin (HbA1C) and ACR, suggesting their potential application in diagnosing DN in T2D subjects (Eissa et al., 2016).

Further research (Delić et al., 2016) detected over 300 miRNAs in a small sample size of DN patients, healthy controls and non-CKD subjects, and singled out miR-320c and miR-6068 as the most upregulated miRNAs. In particular, miR-320c expression showed a significant positive correlation with urinary ACR, and a negative correlation with eGFR. Therefore, urinary exosomal miR-320c was suggested as a possible diagnostic biomarker for DN (Delić et al., 2016). Thrombospondin-1 and -4 (TSP-1; TSP-4) and bone morphogenetic-6 (BMP6), all involved in TGF-β signaling, were revealed as putative miR-320c targets using the miR-Walk database. In the HK2 cell line, BMP6 has been shown to suppress the profibrogenic effects of TGF-β by inhibition of EMT and deposition of matrix proteins and by preventing enhanced adhesion behavior (Yan et al., 2009). TSP-1 has been identified as an activator of TGF-β, found increased in the glomeruli of DN patients, co-expressed with p-SMAD2/3, and its inhibition has resulted in reduced renal impairment and proteinuria (Hohenstein et al., 2008). TSP-4 plays a role in regulating collagen mRNA levels and its deficiency significantly increases cardiac fibrosis (Frolova et al., 2012). Therefore, it could be inferred that miR-320c may be utilized to trace DN, because it regulates TGF-β function and fibrosis progression.

miR-877-3p was identified as a candidate biomarker for DN in two studies: a small-scale study showing it was significantly upregulated in DN compared to T2D (Xie et al., 2017) and a transcriptomic analysis where it was also found to be significantly increased in urinary exosomes from the CKD group compared to healthy controls (Khurana et al., 2017). Predicted targets of miR-877-3p revealed a possible link with cell death, epidermal growth factor receptor signaling, response to hypoxia and to Akt/FoxO signaling (Xie et al., 2018). Specifically, miR-887-3p might accelerate renal dysfunction *via* PI3K/Akt, which in turn leads to the inactivation of the transcription factor FoxO3a and the subsequent downregulation of its proapoptotic genes, consequently inducing TGF-β-mediated mesangial cell survival and oxidative stress in the early DN (Kato et al., 2006).

The differences in miRNAs detected in the various studies are noticeable and may reflect the diverse pathways underlying DN or a lack of reproducibility in uEVs data or different sensitivities of the technologies supporting miRNA evaluation (Table 4).

### 2.2 Autosomal dominant polycystic kidney disease

Autosomal dominant polycystic kidney disease (ADPKD) is an inherited kidney disease caused by mutations in genes PKD1 and PKD2 encoding for proteins polycystin-1 (PC-1) and polycystin-2 (PC-2). PC-1 has features of ion channel and G-protein coupled receptor (GPCR) and PC-2 is a calcium-permeable non-selective cation channel, which in healthy conditions form a complex in the primary cilia that is important for intracellular calcium regulation (Ta et al., 2020). When polycystin function is lost, this in turn leads to a decreased intracellular calcium and increased cyclic adenosine monophosphate (cAMP) levels/protein kinase A activation, activating downstream mTOR signaling responsible for impaired tubulogenesis, cell proliferation, increased fluid secretion and interstitial inflammation (Mise et al., 2018; Chebib et al., 2015). The formation of fluid-filled cysts disrupts the renal parenchyma and often progresses to ESKD (Ong et al., 2015), which in 5%–10% of cases is caused by ADPKD. Although the molecular basis of the disease is known, the complex mechanism of cyst growth and expansion is still little understood. In an initial small-scale clinical study, profiling of a mixed cohort of DN and ADPKD patients led to the identification of osteoprotegerin (OPG) in uEVs and its increased expression in patients with CKD compared to healthy controls (Benito-Martin et al., 2013). OPG is a glycoprotein secreted from proximal TECs that belongs to the tumor necrosis factor (TNF) receptor superfamily involved in the regulation of the skeletal, vascular, and immune system. It is believed to act as a decoy receptor for the death ligand TNF-related apoptosis-inducing ligand (TRAIL) and in DN both OPG and TRAIL appear to be highly expressed (Lorz et al., 2008).

A specific investigation of uEVs proteomics in ADPKD revealed about 30 proteins of which periplakin, envoplakin, villin-1, and complements C3 and C9 were further validated (Salih et al., 2016). Villin-1 is an actin-modifying protein which in kidney is mostly expressed in the proximal tubules, likely to be functionally linked to polycystin-1 as both have a role in cell motility and actin reorganization (Tomar et al., 2006; Castelli et al., 2015). Plakins are transmembrane cytolinker proteins with multiple functions, including participation in the binding of adhesive junctions such as desmosomes (Huber, 2003). Although polycystin-1 is associated with desmosomal proteins, in ADPKD it is no longer colocalized with desmosomes resulting in mispolarization of desmosomal proteins, which may explain the abundant amounts of plakins in patients with ADPKD (Silberberg et al., 2005). The complement system C9 and C3 components are believed to be involved in the progression of ADPKD by mediating cyst-lining epithelial cell proliferation, infiltration of tubulointerstitial inflammatory cells and development of fibrosis. Complement C3 and C9 may be locally produced by cysts epithelial cells and packed in uEVs in patients with ADPKD (Salih et al., 2016). Moreover, inhibition of the complement system results in decreased cyst growth, further confirming its involvement in ADPKD pathogenesis (Wang et al.,2014).

### 2.3 Immunoglobulin A nephropathy and thin basement membrane nephropathy

Immunoglobulin A nephropathy (IgAN) and thin basement membrane nephropathy (TBMN) are characterized by hematuria as a visible or microscopic symptom. Although they are separate kidney conditions the initial clinical presentation may be similar. IgAN, the most common autoimmune type of glomerulonephritis, can manifest from asymptomatic hematuria to rapidly progressive kidney disease. IgAN is due to the presence of galactose-deficient IgAs which are regarded as abnormal by other circulating antibodies, leading to immune complexes which cause inflammation in the glomerulus. In particular, the underlying mechanism involves aberrant glycosylation of IgA1, synthesis of galactose-deficient IgA1 antibodies, formation of immune complexes by binding of the galactose-deficient IgA1 and anti-glycan/glycopeptide antibodies, and finally accumulation of the immune complexes in the glomerular mesangium (Rodrigues et al., 2017; Suzuki et al., 2011). Dysregulation of mucosal IgA production has been reported to cause an inflammatory response to the immune complexes resulting in proliferation of mesangial cells, expansion of ECM, release of cytokines and growth factors, damage to podocytes and tubular cells, all of which led to renal function deterioration (Radford et al., 1997; Lai et al., 2009). The affected patients are characterized by proteinuria and persistent hematuria. Another CKD type characterized by persistent microscopic hematuria but without significant proteinuria is TBMN, an autosomal dominant disorder associated with mutations in type IV collagen genes COL4A3 and COL4A4 leading to reduced thickness and stability of the glomerular basement membrane (GBM) (Badenas et al., 2002; Tryggvason and Patrakka, 2006; Haas, 2006). Although early clinical symptoms are similar for IgAN and TBMN, the outcome is different as IgAN is more severe leading to ESKD in many patients.

A small-scale study has tried to discriminate IgAN and TBMN based on differences in the uEVs proteome (Moon et al., 2011) (Table 3). Compared to healthy controls, over 30 uEVs proteins were found upregulated in IgAN and over 50 proteins upregulated in TBMN. Validation in additional sample cohorts (12 patients with IgAN, 12 patients with TBMN, six healthy volunteers) revealed uEVs vasorin precursor as linked with TBMN, while APN, α-1-antitrypsin and ceruloplasmin as associated with IgAN. Vasorin is a cell-membrane protein, predominantly expressed in vascular smooth muscle cells, that binds directly to TGF-β with its extracellular domain and reduces TGF-β downstream signaling, thereby preventing apoptosis and fibrosis (Ikeda et al., 2004). Interestingly, vasorin was found to be one of the significantly differentially expressed proteins also in a total urinary proteome study of IgAN, being downregulated compared to controls (Samavat et al., 2015). APN is an enzyme catalyzing the removal of basic and neutral amino acid residues from bioactive oligopeptides, which is expressed on the cell brush border membranes of the small intestine, glomerular epithelial and mesangial cells, and it is present in uEVs originating from renal proximal tubules (section 1.5). The involvement of APN in kidney pathologies, mostly *via* binding to its main substrate Ang II is well described (Look et al., 1989; Vlahović and Stefanović, 1998). α-1-antitrypsin, also called Serpin A1, is a member of a superfamily of serine protease inhibitors that targets neutrophil proteases, elastase, cathepsin G, and proteinase 3, but also has moderate affinity for plasmin and thrombin (Clemmensen et al., 2011; Law et al., 2006). α-1-antitrypsin peptides, along with β2-microglobulin fragments, were the most prominent peptides to show a negative correlation with baseline eGFR in CKD (Schanstra et al., 2015).

Exosomal chemokine (C-C motif) ligand 2 (CCL2) was also linked to IgA nephropathy in a study by (Feng et al., 2018). Although the initial group size was small, CCL2 mRNA could effectively distinguish IgAN cohorts from control groups in a validated experiment with a larger cohort of IgAN, MCD, MN and healthy controls, and CCL2 mRNA correlated with the severity of tubulointerstitial fibrosis and C3 deposition in IgAN, with the highest expression of uEVs CCL2 mRNA in patients with >50% biopsy area of tubular atrophy and fibrosis (Feng et al., 2018; Lv et al., 2018).

Among the proposed IgAN uEVs markers, also miRNAs have emerged (Szeto et al., 2019) (Table 4). Digital transcriptomics (NanoString nCounter) initially revealed an array of miRNAs with significantly different expression between IgAN and healthy which upon validation narrowed down to three miRNAs in particular. These were miR-150 which was significantly increased, while miR-204 and miR-555 were significantly decreased in IgAN. Especially, miR-204 displayed the best diagnostic accuracy. Looking at the biological processes involved, it is known that miR-204 antagonizes EMT by targeting specificity protein-1 transcription factor (SP1) in TEC after induction of acute kidney injury protecting renal tubules from chronic fibrotic changes (Chen et al., 2017), therefore it is reasonable to see its change in CKD pathology.

### 2.4 Focal segmental glomerulosclerosis and minimal change disease

Focal segmental glomerulosclerosis (FSGS) and minimal change disease (MCD) are forms of podocytopathies characterized by the appearance of primary lesions of podocytes or visceral epithelial cells. In particular, FSGS is a sclerotic glomerular disease frequently progressing to ESKD and recurrent in ∼25% of kidney transplant patients. They are separate conditions that differ in prognosis and treatment despite the similarities (Cravedi et al., 2013). FSGS can be either primary, secondary (due to viral infection, medication) or genetic and presents tubulointerstitial scarring. The affected glomeruli show segmental solidification of the glomerular tuft. MCD, on the other hand, is characterized by extensive podocyte injury and the presence of normal-looking glomeruli and scar-free tubulointerstitium at the ultrastructural level (Rosenberg and Kopp, 2017).

A comparative study of differentially expressed miRNAs in FSGS and MCD in plasma and urinary EVs (Ramezani et al., 2015) revealed over 150 miRNAs. Among them specific miRNAs could discriminate between the two forms of CKD. miR-1225-5p was validated as upregulated in MCD compared to FSGS and healthy controls, while miR-155 as upregulated in FSGS compared to MCD and controls. miR-1915 and miR-663 were decreased in FSGS compared to MCD and controls, and in addition linked to proteinuria and eGFR changes (Table 4). Previous studies had shown that all these miRNAs are involved in the pathogenesis and progression of renal diseases. In particular, miR-1225-5p and miR-1915 regulate the expression of genes that are involved in maintaining adult renal stem/progenitor cells (ARPCs), for instance key markers of renal progenitors CD133 and PAX2 and genes involved in the repair mechanisms of ARPCs, such as Toll-like receptor 2 (TLR2). Moreover, they are thought to be specifically expressed in ARPCs, and therefore a decrease in their expression levels in the FSGS kidney may potentially reflect the loss of ARPCs (Sallustio et al., 2013). miR-663 has been reported to target renin (*REN*) and apolipoprotein E (*APOE*) mRNAs and a decrease in miR-663 has been associated with an increase in renin mRNA in patients with hypertension (Marques et al., 2011). miR-155 has been reported to target SMAD2 mRNA and repress SMAD2 protein expression, thereby affecting TGF-β-induced SMAD phosphorylation and signaling and altering downstream expression of various genes involved in fibrosis, inflammation and angiogenesis (Louafi et al., 2010).

### 2.5 Lupus nephritis

An autoimmune form of CKD, Lupus nephritis (LN) is a severe glomerulonephropathy that occurs as a frequent complication of systemic lupus erythematosus (SLE), a common type of Lupus. LN occurs as a consequence of the binding of antibodies to multiple intrarenal autoantigens. It can involve the formation of “tertiary lymph follicles” within the kidney including proinflammatory B cells, and the secretion of autoantibodies by plasma cells (Lech, 2013). A serious complication is the appearance of a cellular crescent, consisting of severe active vascular, glomerular and tubulointerstitial lesions, that manifest in up to 50% of patients with LN (Zhang et al., 2016b).

A small size study in LN patients' uEVs revealed elevation of miR-26a, the expression of which concurs to regulate podocyte homeostasis and positively correlates with urinary protein levels (Ichii et al., 2014) (Table 4).

In a parallel study, the presence of active LN versus SLE was associated with uEVs miR-146a (Perez-Hernandez et al., 2015), which also displayed a higher glomerular expression in patients with LN, correlating with disease severity (Lu et al., 2012). Furthermore, an increase in miR-335*, miR-302d, highly abundant in human urine (Weber et al., 2010), and miR-200c, was associated with SLE (Perez-Hernandez et al., 2015). miR-200c was previously reported to discriminate patients with acute kidney injury from healthy individuals in urine (Ramachandran et al., 2013).

In a further small-scale study, the urinary exosomal miRNA expression profiles in patients with type IV lupus nephritis (LNIV), patients with LNIV with cellular crescent (LNIV-CC), and healthy subjects were analyzed by high-throughput miRNA sequencing (Li et al., 2018). Validations revealed that miR-3135b was significantly enhanced in LNIV, while miR-654-5p was significantly enhanced in LNIV-CC. miR-146a-5p (found in LNIV by Perez-Hernandez et al., 2015) did not show a significant difference in expression between LNIV-CC and LNIV cohorts (Li et al., 2018). A link between miR-3135b or miR-654-5p with kidney disease was not previously reported, but miR-3135b had been associated with dilated cardiomyopathy (Wang et al., 2017), and miR-654-5p with prostate cancer cell proliferation (Östling et al., 2011).

Among all these uEVs studies in LN, a multi-marker urinary exosomal panel emerged as a stronger approach to the early detection of LN (Solé et al., 2019) and for identifying ESKD risk. Three miRNAs (miR-21, miR-150, and miR-29c) were found to correlate with the chronicity index in the renal biopsy, *via* inducing profibrotic changes such as formation of collagen (COL4A1) and potentially targeting the VEGFA and SP1 genes (Solé et al., 2019) leading to activation of pSMAD2/3 and the coactivator p300, and progression of glomerulonephritis (Kassimatis et al., 2010).

### 2.6 Pediatric nephrotic syndrome

Pediatric nephrotic syndrome (NS) is a chronic childhood glomerular disease associated with glomerular podocyte dysfunction due to primary T-cell disorder. It is usually preceded by MCD and FSGS but can also be caused by genetic disorders or be secondary to other diseases such as infections and neoplasia (Eddy and Symons, 2003).

Five potential miRNA biomarkers for pediatric NS were detected by high-throughput Illumina sequencing followed by RT-qPCR validation (Chen et al., 2019). The expressions of miR-194-5p, miR-146b-5p, miR-378a-3p, miR-23b-3p, and miR-30a-5p were significantly increased in pediatric NS compared to healthy control samples, and remarkably reduced during the period of clinical remission. Among them, uEVs miR-194-5p, and miR-23b-3p had the highest diagnostic accuracy and could discriminate between high proteinuria and low proteinuria, hence predict the severity of pediatric NS (Chen et al., 2019). miR-194-5p was also reported in uEVs of patients with early DN (Jia et al., 2016) (Section 2.1). The miR-146 family was also involved in LNIV as commented in Section 2.5 (Perez-Hernandez et al., 2015), while miR-378a-3p targets glomerular matrix protein nephronectin (NPNT) decreasing its levels (Müller-Deile et al., 2017). Moreover, miR-23b-3p and miR-30a-5p are specific in exosomes and enriched in human urine (Cheng et al., 2014). Therefore, the individual miRNAs of this panel were not totally unexpected as they did recur in the literature as either present in uEVs or linked with kidney pathology.

### 2.7 All-purpose markers of CKD

UEVs proteomics and transcriptomics have also been utilized to identify generic markers of CKD. An early investigation, on biopsy-proven pools of CKD patients including DN, FSGS and IgAN, identified miR-29 and miR-200 families as downregulated in uEVs of patients with CKD (Lv et al., 2013). In terms of biological significance, *in vitro* and *in vivo* studies had shown downregulation of miR-29 in conditions of renal fibrosis and both miR-29 and miR-200 respectively targeted TGF-β1-induced production of collagens I and III (Qin et al., 2011), ZEB1/2 and fibronectin. Hence it is logical that their downregulation may be pro-fibrotic (Tang et al., 2013). A generic marker of CKD may be CD2-associated protein (CD2AP). CD2AP is expressed in the podocyte foot processes in the glomerulus, helping to keep the integrity of the slit diaphragm *via* interactions with other podocyte proteins (Kravets and Mallipattu, 2020). The transcript of CD2AP was found to be reduced in the uEVs of CKD patients with heavy proteinuria compared to moderate and mild proteinuria patients indicating a negative correlation of CD2AP with proteinuria severity (Lv et al., 2014).

Furthermore, in the attempt to relate non-coding RNAs to CKD stages, miR-181a emerged as 200-fold reduced in expression in all four stages of CKD compared to healthy controls (Khurana et al., 2017). As circulating miR-181a from human serum was significantly increased in samples of patients with nephrotic syndrome (Sui et al., 2014), a reduced level of miR-181a in uEVs (Khurana et al., 2017) appears a logical finding.

When miRNAs previously linked to CKD such as miR-21, miR-30a-5p and miR-92a (Baker et al., 2017; Zhang et al., 2014; Henique et al., 2017) were searched in the uEVs of patients with CKD arising from different aetiologies (Lange et al., 2019), only miR-21 resulted significantly different (upregulated) between CKD patients and healthy controls, showing a negative correlation with eGFR (Lange et al., 2019). A further study revealed that miR-21-5p was increased and miR-30b-5p downregulated in patients with diabetic and non-diabetic CKD compared to controls. Although they cannot be regarded as specific biomarkers of DN, their dysregulation in expression may be linked with renal impairment (Zang et al., 2019).

In more recent investigations, when a CKD cohort was divided into diabetic and non-diabetic subgroups, miR-451 emerged as significantly higher in the non-diabetic CKD subgroup and to negatively correlate with eGFR decrease (Kumari et al., 2020). In primary human proximal tubule cells miR-451 knockdown resulted in enhanced expression of downstream mRNA targets, including tyrosine 3-monooxygenase/tryptophan 5-monooxygenase activation protein zeta (YWHAZ) (Kumari et al., 2020), which in DN downregulates p38 MAPK signaling, with inhibition of glomerular mesangial cell proliferation. Therefore, it was proposed that miR-451 may play a protective role in early DN (Zhang et al., 2012).

### 2.8 uEVs protein and miRNA with proposed prognostic value in CKD

CKD stratification markers and early markers of disease progression are much needed as currently CKD is diagnosed by means of a kidney biopsy which can confirm fibrosis only at the point when it is already well established and advanced. At this stage the risk for the patient to progress to ESKD is high and part of the kidney function is lost. Only few studies have focused on CKD stratification markers, such as a study by (Musante et al., 2015) which has relied on uEVs protease and protease inhibitors to stratify DN patients. Several proteases were found differently expressed in the uEVs of the DN cohort, of which cathepsins of various classes, kallikrein-13, and proteinase-3 were the most significantly altered. Among the protease inhibitors, uEVs cystatin B was the most significantly changed in all DN groups compared to healthy subjects, but also linked to normoalbuminuria. While uEVs cathepsins were the most increased in the micro-macroalbuminuric groups, kallikrein 13 and proteinase-3 were most expressed in the norm-microalbuminuric groups with a decrease in the macroalbuminuric group (Musante et al., 2015). Hence this set of uEVs proteins potentially allow DN patient stratification based on the degree of albuminuria.

C-megalin (gp330), a large membrane glycoprotein of a low density lipoprotein receptor (LDLR) family (Saito et al., 1994) has also been shown to be associated with albuminuria progression when identified in uEVs from DN subjects. C-megalin correlates with ACR, and negatively correlates with eGFR (De et al., 2017). Advanced glycation end products and fatty acid-enriched proteins have been identified as toxic megalin ligands, which are filtered by glomeruli and once reabsorbed cause proximal TEC injury (Kuwahara et al., 2016).

Nephronophthisis-related ciliopathies (NPH-RC) are a group of autosomal recessive cystic kidney diseases associated with impairments in the structure and function of the primary cilia, a common genetic cause of ESKD (Choi et al., 2019). Stokman et al. (2019) studied uEVs proteins in patients with NPH-RC and found several proteins differently expressed at different stages of CKD and healthy controls. In particular, dipeptidase 1 (DPEP1), an enzyme located in the proximal convoluted tubules responsible for dipeptide hydrolysis (Nafar et al., 2014), and protocadherin Fat 4 (FAT4), present in cilia in kidney cells with a key role in the polarity of planar cells (Saburi et al., 2008), were downregulated in uEVs from the CKD cohorts. Versican core protein (VCAN), an extracellular matrix proteoglycan normally expressed in the kidneys at low levels (Rudnicki et al., 2012), was upregulated in uEVs of CKD cohorts. The expression of these discriminating proteins correlated with the severity of CKD so that they were most decreased (DPEP1 and FAT4) and increased (VCAN) in the advanced stage of CKD (Stokman et al., 2019).

It has been recently reported that AQP1 and AQP2, water channels also found in uEVs (Pisitkun et al., 2004), become reduced in uEVs during advanced CKD (Oshikawa-Hori et al., 2021). Comparison of different stages of CKD have shown that AQP1 and AQP2 are significantly reduced in stages 4 and 5 of CKD at a high level of diagnostic accuracy (Oshikawa-Hori et al., 2021), which increases when uEVs AQP and AQP2 are considered in combination as markers of advanced CKD.

**FIGURE 3:**
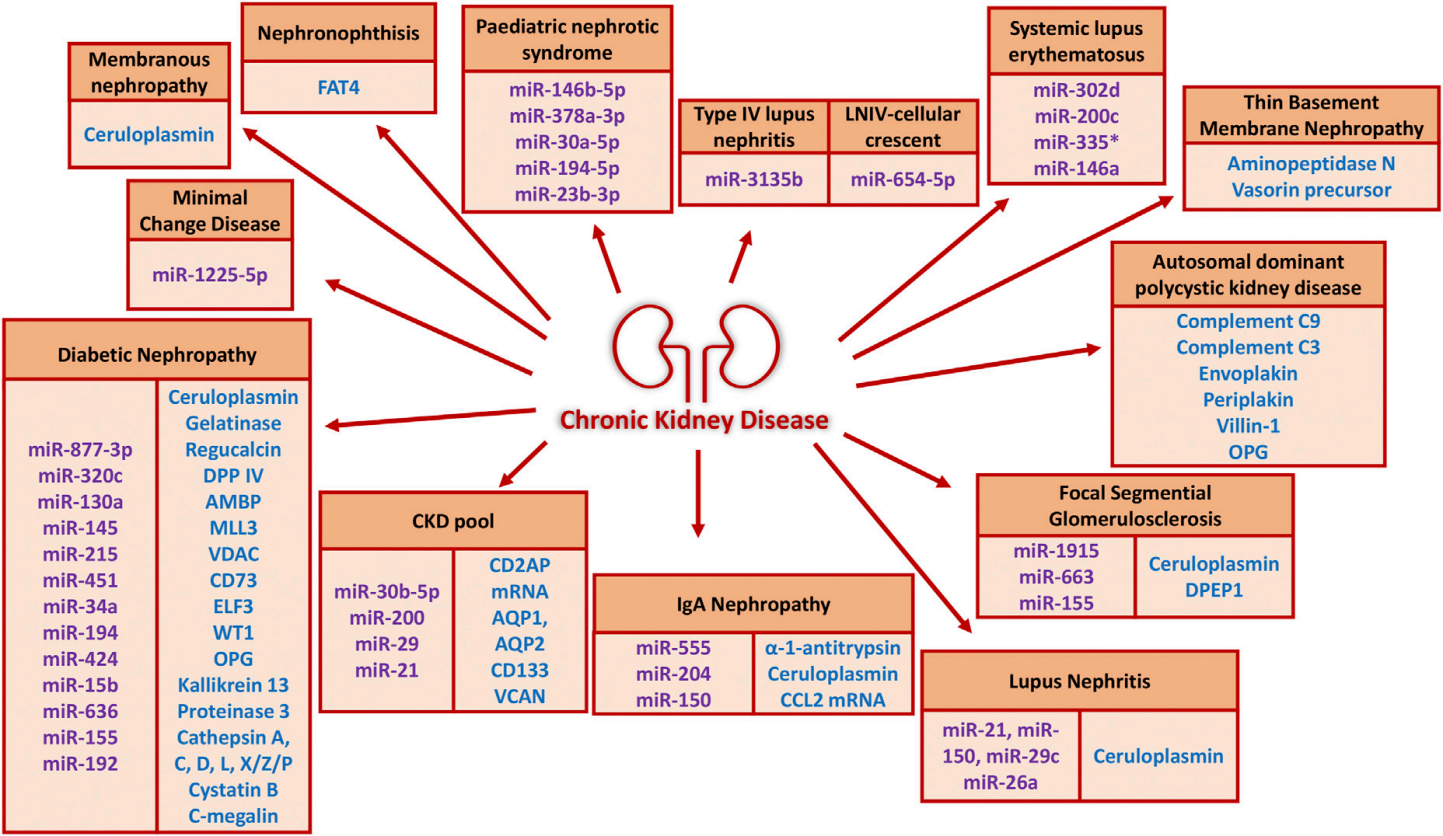
uEVs proteins and miRNAs of potential diagnostic and prognostic value in the literature (since 2012) grouped according to CKD etiologies. The protein cargo is shown in blue and the miRNA cargo in purple.

All the markers discussed in this section have been combined and listed in Figure 3.

## 3 Urine handling and storage

Despite urine being a valuable source of biomarkers from the urogenital tract, lack of consensus for standardization of urine collection, processing, handling and storage, as well as uEVs isolation and downstream analysis, may be responsible for the high inter-individual sample variability, hampering the discovery of reliable biomarker candidates (Barreiro et al., 2021). Information on collection, storage, and timing of uEVs isolation for the reviewed studies is summarized in Table 5. Over 90% of the reviewed studies provide access to information on urine sample collection and processing protocols. Approximately 30% of the studies relied on first-morning or early-morning urine, 15% second-morning urine, and few others reported mid-day urine or overnight urine collection. In over 30% of the studies urine samples were treated with protease inhibitors. The effect of collection timing on uEVs and uEVs biomarkers has been reported by Zhou et al., 2006 showing that the total amount in uEVs protein was similar between first and second-morning urine, and that four tested exosomal proteins remained constant. This study suggests that both first and second morning urine collections can be used for uEVs isolation and protein analysis (Zhou et al., 2006). In most studies, urine samples were centrifuged to remove cells and cell debris, however a wide variation in centrifugal speeds and times was reported (Table 5), with the most frequent centrifugation set at 2,000xg. Regarding the timing of uEVs isolation, in about half of the studies uEVs were isolated immediately after urine collection, while in the other half of the studies urine was stored for later uEVs isolation and further analysis at a temperature ≤80°C. There are few reports linking uEVs quality to storage temperature, showing that freezing uEVs at −20°C results in greater loss in uEVs proteome, while freezing at −80°C preserved above 85% of specific uEVs proteins (Zhou et al., 2006). A more recent study reported that storing uEVs at −20°C reduces RNA and protein yield compared to −80°C, although uEVs morphology is not affected (Barreiro et al., 2021). Therefore, storing uEVs at −80°C appears to be critical for the successful uEVs conservation and subsequent analysis of the uEVs cargo. Details on uEVs storage length are hardly provided in the literature.

**TABLE 5:** Urine handling and storage conditions as reported in the studies included in this review.

Urine collection	Spot urine (Kalani et al., 2013; Zang et al., 2019; Kumari et al., 2020)	First/early-morning urine (Lv et al., 2013; Lv et al., 2014; Musante et al., 2015; Perez-Hernandez et al., 2015; Jia et al., 2016; Xie et al., 2017; Feng et al., 2018; Li et al., 2018; Szeto et al., 2019; Cappelli et al., 2020)	Second-morning urine (Benito-Martin et al., 2013; Zubiri et al., 2014; Salih et al., 2016; De et al., 2017)	Unspecified morning urine (Moon et al 2011; Chen et al., 2019; Lange et al., 2019; Dimuccio et al., 2020)	Not the first morning urine (Stokman et al., 2019)	Mid-day urine (Ramezani et al., 2015)	Voided urine (Eissa et al., 2016)	Overnight urine collection (Barutta et al., 2013)	Unspecified (Sun et al., 2012; Ichii et al., 2014; Gudehithlu et al., 2015; Zubiri et al., 2015; Delić et al., 2016; Khurana et al., 2017; Sakurai et al., 2019; Gudehithlu et al., 2019; Solé et al., 2019)
Timing of uEVs isolation	Right away (on sample collection) ∼ 47% of studies	After thawing ∼ 47% of studies	Unspecified (Ichii et al., 2014; Khurana et al., 2017)						
Centrifugation to remove cell debris	300 × g (10 min at RT or 15 min at 4°C) (Benito-Martin et al., 2013; Jia et al., 2016; Sakurai et al., 2019)	300 × g (10 min) then 17,000 × g (20 min) at 4°C (Barutta et al., 2013)	500 × g (10 min); then 2,000 × g (20 min) at 4°C (Stokman et al., 2019)	1,000 × g (15–20 min) at 4°C (Gudehithlu et al, 2015; Gudehithlu et al., 2019)	2,000 × g (10–15 min) (Ramezani et al., 2015; Delić et al., 2016; Xie et al., 2017; Zang et al., 2019)	2,000 × g (20–30 min) (Lv et al., 2013; Lv et al., 2014; Musante et al., 2015; Feng et al., 2018)	2,250 × g or 2,500 × g (30 min) (Perez-Hernandez et al., 2015; Li et al., 2018)	3,000 × g (10 min) at RT (Chen et al., 2019)	3,000 × g for 30 min; then 13,000 × g (5 min) at 4°C (Szeto et al., 2019)
Addition of protease inhibitors	Described ∼ 35% of the studies	Unspecified ∼ 62% of studies	Not used (Musante et al., 2015)						
Storage condition of urine/uEVs	∼ −80°C Most studies (94%)	Unspecified (Ichii et al., 2014; De et al., 2017)							

## 4 Conclusion and future perspectives

Urinary EVs are a precious source of proteins and miRNAs, which although scarcely present in soluble form in full urine, are concentrated in EVs and discoverable through uEVs proteomics and transcriptomics. Since 2012, several proteins and individual miRNAs which are uEVs cargo have been proposed as promising CKD biomarkers. Our review has reported 33 different proteins and 37 different RNA in uEVs selected among the most relevant to CKD. The criteria was to select those molecules emerged from unbiased often “big data” screening but validated through a second targeted approach and ideally also confirmed in larger cohorts. The “hunt” for uEVs markers started approximately a decade ago and has taken place for most CKD types up to now. However, studies are far from uniform both in terms of CKD stage or eGFR and the way urine samples are collected, stored and processed to isolate EVs. Either because of the intrinsic variability of uEVs composition in the individual subjects, or depending on the urine collection timing and storage conditions, or because CKD classifications have also loose margins and overlap, a huge number of different molecules have emerged as potential disease markers. A selection of uEVs proteins such as gelatinase and ELF3 in DN, plakins (periplakin, envoplakin) and complements (C3 and C9) in ADPKD, and ribonucleic acids such as CCL2 mRNA in IgAN, miR-194-5p and miR-23b in pediatric NS have been linked to CKD severity and therefore they may have usefulness as stratification markers if not a potential prognostic value. There is a great variety of miRNA proposed. A bunch of miRNAs (miR-21, miR-150, and miR-29) have been reported to better mirror CKD in combination rather than individually, and together with miR-155, miR-194 are recurrent in different uEVs biomarkers discovery studies. Among the potential uEVs protein biomarkers, only ceruloplasmin was detected in more than one CKD study, associated with several kidney diseases, and as such uEVs ceruloplasmin may be regarded as a general marker of CKD pathology. Not many studies have compared the effectiveness of biomarkers detection from uEVs and full unfractionated urine, however uEVs ceruloplasmin has emerged as a better marker for early kidney disease.

A benefit of uEVs markers is that they generally better reflect early changes in the kidney than full urine and as such precede proteinuria. This is a key point that warrants future scrutiny, especially as new tools allowing direct uEVs marker quantifications in biofluids without necessity to isolate uEVs have emerged and are available to the scientific community (e.g. Exoview platform, Tonoli et al., 2022).

Attempts to relate the selected uEVs CKD markers to the molecular cell biology process underlying disease development have been reviewed or suggested in our evaluation. Future research in this direction may lead to better insights on the pathology and help reveal potentially new mechanisms as well as anti-fibrotic targets. The uEVs cell origin is particularly informative as it allows to track the molecular and cellular events underlying the CKD condition predicting the disease evolution. Although much effort has been made to identify specific cell markers in uEVs, this area is still not well consolidated and again uEVs markers quantifications directly in the biofluids should accelerate progress. Tracking the cell origin of uEVs by means of spatial transcriptomics is also a potential way to build the larger picture of CKD progression. Lastly, despite considerable effort by many groups globally, all the studies reviewed here included less than 1000 CKD patients when considered together, of which half were DN. Larger size clinical studies should address these key points to reveal the full usefulness of the uEVs approach over full urine tests, and as such accelerate translation of uEVs biomarker discoveries into clinical practice to offer a real alternative to kidney biopsy.
